# Communicating Bad News in Emergency Health Care: clinicians needs and perceptions - a comparative study between Portuguese and Brazilian samples

**DOI:** 10.1192/j.eurpsy.2025.1859

**Published:** 2025-08-26

**Authors:** C. Pires-Lima, F. Bittencourt-Romeiro, S. Morgado-Pereira, M. Figueiredo-Braga

**Affiliations:** 1 Neurosciences and Mental Health Department; 2 CINTESIS – Center for Health Technology and Services Research, Faculty of Medicine, University of Porto, Porto, Portugal; 3 Faculdade de Tecnologia, SENAI, Porto Alegre - Rio Grande do Sul, Brazil; 4 IVAR – Research Institute for Volcanology and Risk Assessment, University of the Azores, Ponta Delgada, Azores; 5 i3S – Institute for Research and Innovation in Health, University of Porto, Porto, Portugal

## Abstract

**Introduction:**

Communicating bad news is a common occurrence in the healthcare sector, especially in emergency services. However, it is also recognized as one of the most stressful, uncomfortable and difficult moments. Clinicians may appear cold, exhibiting depersonalized communication, or feel detached and overwhelmed due to a lack of training in communicating bad news. Therefore, it is crucial to develop their skills in communicating bad news to improve their proficiency in these challenging situations.

**Objectives:**

The purpose of this study is to comprehend the specific needs of clinicians when delivering bad news to emergency patients, and to identify effective strategies for tailoring educational programs to the demands of emergency healthcare. Additionally, the study aims to identify differences and similarities across two geographic and cultural settings.

**Methods:**

A group of emergency health professionals working in Portugal and Brazil were invited to participate in the study. We will collected sociodemographic data and performed a professional characterization. Participants will describe their experience and training in delivering bad news in emergency settings and preferences regarding methods and design of communication skills training. The evaluation included clinicians self-evaluate their knowledge, skills, and application of seven relevant skills (adapted from Breaking Bad News, Servotte et al 2019).

**Results:**

We expect clinicians from emergency health units in Portugal and Brazil to identify specific strategies applied when delivering bad news, and how theoretical knowledge and previous training imbed their sense of capability. Sociodemographic and professional characteristics are probable factors influencing clinicians’ self-perception of communicating bad news to emergency patients and families.

**Conclusions:**

Breaking bad news can be challenging for clinicians due to the complexity of communication and the emotional intensity involved. This highlights the need for tailored training programs that are culturally adapted and focused on clinicians’ needs and preferences. Albeit speaking the same language differences in the healthcare setting (Portugal vs. Brazil) must be considered when designing educational interventions.

**Disclosure of Interest:**

None Declared

